# The novel mitochondria localization of influenza A virus NS1 visualized by FlAsH labeling

**DOI:** 10.1002/2211-5463.12336

**Published:** 2017-11-06

**Authors:** Chuan‐Fu Tsai, Hsin‐Yi Lin, Wei‐Li Hsu, Ching‐Hsiu Tsai

**Affiliations:** ^1^ Graduate Institute of Biotechnology National Chung Hsing University Taichung Taiwan; ^2^ Graduate Institute of Microbiology and Public Health National Chung Hsing University Taichung Taiwan

**Keywords:** influenza A virus, mitochondria localization, NS1 protein, tetracysteine‐tag

## Abstract

The nonstructural protein 1 (NS1) of the influenza A virus (IAV) is a multifunctional protein that counteracts host cell antiviral responses and inhibits host cell pre‐mRNA processing. NS1 contains two nuclear localization signals that facilitate NS1 shuttling between cytoplasm and nucleus. In this study, we initially observed the novel mitochondria localization of NS1 in a subset of transfected cells. We then further monitored the localization dynamics of the NS1 protein in live cells infected with IAV expressing NS1 with insertion of a tetracysteine‐tag. The resulting mutant virus showed similar levels of infectivity and expression pattern of NS1 to those of wild‐type IAV. Pulse labeling using a biarsenical compound (fluorescein arsenical hairpin binder) allowed us to visualize the dynamic subcellular distribution of NS1 real time. We detected NS1 in mitochondria at a very early infection time point [1.5 h postinfection (hpi)] and observed the formation of a granular structure pattern in the nucleus at 4 hpi. This is the first identification of the novel mitochondria localization of NS1. The possible role of NS1 at an early infection time point is discussed.

**Abbreviations**
DAPI: 4′, 6‐diamidino‐2‐phenylindole
DMEM: Dulbecco's modified Eagle's medium
EMEM: Eagle's minimum essential medium
FlAsH: fluorescein arsenical hairpin binder
HEK 293: human embryonic kidney 293
hpi: hour postinfection
IAV: influenza A virus
IFA: immunofluorescence assay
IFN: interferon
MAVS: mitochondrial antiviral‐signaling protein
MDCK: Madin–Darby canine kidney
MOI: multiplicity of infection
NS1: nonstructural protein 1
PI3Ks: phosphatidylinositol 3‐kinases
RIG‐I: retinoic acid‐inducible gene I
tc: tetracysteine
WT: wild‐type

All influenza A viruses (IAV) possess single‐stranded negative polarity of eight RNA segments 1. The eight viral RNAs encode at least 11 known proteins. The intron devoid of mRNA of influenza virus encodes six RNA segments – PB2, PB1, PA, HA, NP, and NA proteins. The nonstructural protein 1 (NS1) and the M1 proteins are encoded by viral mRNA, which contain introns that do not undergo splicing of viral RNA segments seven and eight. By contrast, the spliced mRNA of viral RNA segments seven and eight encode the M2 and the NEP/NS2 proteins, respectively 2, 3. Some strains of IAV also express the 11th protein, PB1‐F2, which has been shown to be encoded by an alternate open reading frame near the 5′‐end of the PB1 gene 4. In recent times, two truncated NS1 proteins translated from downstream AUG were identified 5. As with the full‐length NS1, the N‐truncated NS1 proteins enhance suppression of interferon (IFN) productions.

The entry of influenza virus has been shown by conventional clathrin‐mediated endocytosis 6; however, a nonclathrin, noncaveolae‐mediated internalization mechanism has also been observed 7. Following endocytosis, the complete uncoating process is the HA‐mediated fusion of the viral membrane with the endosomal membrane and the M2‐mediated release of the RNP complexes in the cytoplasm 8. The half‐time for viral penetration is about 25 min after adsorption. Approximately 10 min later, RNP complexes are found in the nucleus 8. The RNP complexes are transported through nuclear pores into the nucleus, and then, transcription and replication of viral RNA takes place 9.

Nonstructural protein 1 is not a structural component of the virion, but expressed at high levels in infected cells 10. NS1 protein is dissected into an N‐terminal RNA‐binding domain 11 and a C‐terminal effector domain 12. Results of X‐ray structure indicate that, in the presence of dsRNA, the full‐length NS1 protein can form the tubular structure 13. NS1 protein predominantly localizes in the nucleus 14, but is found in the cytoplasm at a later time in the infected cells 15, 16. NS1 has been demonstrated to block the functions of two cytoplasmic antiviral proteins, 2′‐5′‐oligoadenylate synthetase 17 and dsRNA‐dependent serine/threonine protein kinase R 18, by RNA‐binding activity of its RNA‐binding domain.

To visualize the intracellular locations of interesting proteins, immunofluorescence assay (IFA) with antibody labeling and detection is mostly performed. This method is easy and flexible, but information gained from this technique is limited due to its end‐point analysis. Real‐time analysis of protein dynamics in live cells is crucial to understand in‐depth protein biochemical and structural changes related to cell behavior and function 19. The most frequent strategy used in the study of detection and analysis of proteins in live cells is the fusion of fluorescent proteins such as GFP with target proteins 20. However, the argument has arisen that the large‐molecule GFP (about 28 kDa) may interfere with the activity, localization, or conformation of its fusion partner. Another disadvantage is that the position of the fluorescent proteins for fusing with the target protein is limited to the N or C terminus. Lately, another technique developed for specifically labeling proteins in live cells has been reported by using the high affinity with tetracysteine (tc) motif, consisting of two cysteine pairs separated by two amino spacers (CCXXCC), and a biarsenical compound under reducing condition 21. One of the biarsenical compounds, fluorescein arsenical hairpin binder (FlAsH), is a membrane‐permeable dye and fluorescently active only upon binding with the TC motif. The dynamic imaging of HIV‐1 Gag protein 22 and Ebola virus protein VP40 23 inside the cells has been demonstrated. Furthermore, dynamic tracking of virus particles by labeling FlAsH with viral protein has been reported in viruses such as HIV 24, flock house virus 25, and vesicular stomatitis virus 26. Recently, recombinant tc‐tagged NS1 IAV has been described and the nuclear localization of NS1 was reported 27. This system has been utilized for correlative electron microscopy 28, fluorophore‐assisted light inactivation 29, 30, and pulse‐chase experiments 28 as well as the fluorescence detection of proteins in live cells.

Nonstructural protein 1 has been shown to interact with a number of cellular factors 17, 18, 31, 32, 33, 34, 35, 36. It is of interest to explore whether such interactions influence the NS1 cellular localization, which may in turn contribute to the versatile functions of NS1. In addition, the localization pattern of NS1 may be influenced by virus strains 37 and duration of virus infection 16. Hence, it is likely that some of the viral and host factors play key roles in determining the intracellular localization of the NS1 protein. However, the artifact resulting from fixation procedure 12 indicates that the localization of NS1 simply determined by conventional immunofluorescent assay may involve some bias. Therefore, an attempt was made to monitor localization dynamics of NS1 in real time during virus infection. To this end, we generated a recombinant influenza virus containing a tc‐tag in NS1 and optimized the FlAsH labeling protocol. The novel dynamic localization of NS1 was detected in the course of virus infection in live cells after FlAsH labeling.

## Materials and methods

### Cells and viruses

Madin–Darby canine kidney (MDCK) cell line (BCRC 60004), human embryonic kidney 293 cell line (HEK 293), and HEK 293T (HEK293 transformed with large T antigen) cell line were maintained in Eagle's minimum essential medium (EMEM; HyClone, Logan, UT, USA) and grown in Dulbecco's modified Eagle's medium (DMEM; GIBCO, Grand Island, NY, USA), respectively, supplemented with 1.0 mm sodium pyruvate, 1.5 g·L^−1^ sodium bicarbonate, 1 unit·mL^−1^ penicillin G sodium, 100 μg·mL^−1^ streptomycin sulfate, and 10% fetal bovine serum (GIBCO). Human influenza virus A/Puerto Rico/8/34 H1N1 (PR8) used in this study was kindly provided by L. Tiley (Department of Veterinary Medicine, University of Cambridge, UK).

### Plasmids and constructs

To generate plasmid expressing NS1 with S‐tag, the full‐length NS cDNA clone pRF486 was used as the template 38. The NS1 was amplified by PCR using the primer sets 5′‐GATGAAAGAAACCGCTGCTGCTAAATTCGAACGCCAGCACATGGACAGCATGGATCCAAACACTGTGTC‐3′ (sequences of S‐tag, underlined) and 5′‐GTCAAACTTCTGACCTAATTG‐3′. To construct the plasmid harboring the NS1 with insertion of tc‐tag at the linker region, two sets of primers were designed for PCR to generate the two halves of NS1 and overlapped each other on the inserting tc sequence: the 5′ half using forward primer FluNS1‐*Hin*dIII‐16–36‐F (5′‐GTGTCAAGCTTTCAGGTAGA‐3′) and reverse primer H1N1‐NS1‐218–237‐tc‐tag(−) (5′‐ACAGCAGCCCGGACAGCACATTTTAAGTGCCTCATCGG‐3′); the other half using forward primer H1N1‐NS1‐238–257‐tc‐tag(+) (5′‐TGCTGTCCGGGCTGCTGTACCATGGCCTCTGTACCTGC‐3′) and reverse primer FluNS1‐pRF486‐*Apa*I‐R (5′‐GACCAGAGGGCCCCGGGCGC‐3′). For both PCRs, plasmid pRF486 harboring full‐length NS gene was used as the DNA template 38. The PCR products were then used as new templates by base pairing each other after denaturation and annealing with the overlapped region at the tc‐tag sequence for the further PCR cycles with the primer set FluNS1‐*Hin*dIII‐16–36‐F and FluNS1‐pRF486‐*Apa*I‐R. The final products were cloned into pGEM‐T Easy vector (Promega, Madison, WI, USA) and sequenced to ensure plasmid pGEM‐NS1‐tc‐linker with the correct sequence for further study. The plasmid pGEM‐NS1‐tc‐linker was digested with the enzymes *Hin*dIII and *Apa*I (New England Biolabs, Beverly, MA, USA) and replaced the corresponding region of plasmid pRF486 with the same enzyme sites, resulting in the plasmid pRF486‐NS1‐tc‐linker. Another mutant pRF486‐NS1‐tc‐ED containing NS1 gene with tc‐tag at effector domain was constructed following the similar strategy. The two sets of primers were FluNS1‐*Hin*dIII‐16–36‐F/H1N1‐NS1‐347–369‐tc‐tag(−) (5′‐ACAGCAGCCCGGACAGCAGATCGCCTGGTCCATTCAGATAC‐3′) and H1N1‐NS1‐370–390‐tc‐tag(+) (5′‐TGCTGTCCGGGCTGCTGTATGGATAAGAACATCATACTG‐3′)/FluNS1‐pRF486‐*Apa*I‐R.

### Transfection

Cells were grown on 13‐mm glass coverslips at 70–80% confluency in 12‐well plates. Transfection was performed with Lipofectamine 2000 according to the manufacturer's instruction (Invitrogen, Carlsbad, CA, USA). Briefly, plasmid (1.5 μg) was diluted in 50 μL serum‐free DMEM and was then mixed with 4 μL of Lipofectamine 2000 diluted in 50 μL serum‐free DMEM. After incubation at room temperature for 40 min, the DNA/liposome mixture was overlaid onto cell monolayers for 14 h, till further analysis.

### Reverse genetic system and virus infection

Recombinant PR8 influenza virus was generated by transfection of plasmids, pRF507 (PB2), pRF508 (PB1), pRF509 (PA), pRF489 (HA), pRF484 (NP), pRF512 (NA), pRF513 (M), pRF486 (NS), pHMG‐PB1, pHMG‐PB2, pHMG‐PA, and pHMG‐NP, which were a kind gift from R. Fouchier (National Influenza Center and Department of Virology, Erasmus, the Netherlands), into 293T cells. The plasmids were prepared according to the manufacturer's instruction of plasmid miniprep purification kit DP01MD‐20 (GeneMark, Tainan, Taiwan) to remove endotoxin. Reverse genetics were conducted to generate the recombinant influenza viruses. Briefly, about 10 μL of Lipofectamine 2000 (Invitrogen) was added to 200 μL serum‐free DMEM and incubated at room temperature for 40 min. The DNA mixtures containing 666 ng each of plasmids were added into 200 μL serum‐free DMEM/Lipofectamine mixture and incubated at room temperature for 15 min. Trypsinized 293T cells were resuspended in complete DMEM at the density of 10^6^ mL^−1^ and seeded with 1 mL for each transfection into a six‐well plate. The DNA/Lipofectamine mixture was dripped onto cells gently after 15‐min incubation and cultured overnight at 37 °C. Medium was then carefully changed with 2 mL of serum‐free DMEM with 0.14% BSA and 1 μg·mL^−1^ trypsin (Worthington Biochemical Co., Lakewood, NJ, USA). After a 2‐day incubation, the virus particles contained in the medium were harvested by centrifugation at 3000 ***g*** for 5 min to remove cells. The clarified supernatant was transferred to microfuge tubes and stored at −80 °C and labeled as P0. MDCK cells were infected with P0 virus in serum‐free EMEM containing 1 μg·mL^−1^ trypsin, and then, the medium was harvested and the cytopathic effects were examined. Finally, the P1 virus in the harvested medium was stored at −80 °C.

### Plaque assay

To determine the virus titer, MDCK cells were grown as a monolayer on 12‐well plates and then inoculated with 10‐fold serial dilution of wild‐type (WT) or mutant viruses in EMEM containing 1 μg·mL^−1^ trypsin for 1 h at 37 °C. Cells were then overlaid with EMEM containing 0.6% UltrapureTM agarose (Invitrogen) and 1 μg·mL^−1^ trypsin at 37 °C for 72–96 h. The infected cells were fixed in methanol and stained with 1% Crystal Violet (Sigma‐Aldrich, St. Louis, MO, USA). The plaques were visualized and counted.

### Immunoblotting

Madin–Darby canine kidney cells were infected with WT or mutant viruses at a multiplicity of infection (MOI) of 1 at 37 °C. At 18 h postinfection (hpi), cells were lysed in 1× lysis buffer [20 mm Hepes, pH 8.0, 25% (v/v) glycerol, 0.5 m NaCl, 1.5 mm MgCl_2_, 0.2 mm EDTA, pH 8.0] containing protease inhibitors (Roche Diagnostics GmbH, Mannheim, Germany) by frozen/thawed method and clarified by centrifugation at 16 100 ***g*** for 10 min at 4 °C. The cellular extract was mixed with the sample buffer, boiled for 5 min, and analyzed by SDS/PAGE on a 12% gel. To detect the specific viral protein on a western blot analysis, total proteins were transferred onto nitrocellulose membranes (PerkinElmer, Waltham, MA, USA). The membranes were then blocked with skimmed milk in TBS buffer, incubated with anti‐NS1 IgY raised by immunizing chicken with recombinant NS1 protein or anti‐NP serum (ab20343; Abcam, Cambridge, UK) in MTBS buffer with 0.1% Tween 20 (Sigma) for 2 h, followed by incubation with horseradish peroxidase‐conjugated rabbit anti‐chicken IgY secondary antibody (1 : 10 000; Thermo Fisher Scientific, Taipei, Taiwan) in MTBS buffer with 0.1% Tween 20 for 1 h. Proteins were detected by a Novex® ECL HRP chemiluminescent substrate reagent kit (Invitrogen) with Kodak image station 2000MM system.

### Immunofluorescence

For indirect immunofluorescence analysis, cells were fixed with 4% paraformaldehyde (Serva Electrophoresis GmbH, Heidelberg, German) for 10 min at room temperature, washed with PBS twice, and permeabilized in 0.1% Triton X‐100 (Merck, Kenilworth, NJ, USA) in PBS for 10 min. After washing with PBS twice, cells were then treated with Image‐iTTM FX signal enhancer (Molecular Probes, Inc., Eugene, OR, USA) for 30 min and washed again with PBS twice. Subsequently, cells were incubated with primary antibodies, such as anti‐S‐tag antibody (1 : 2000; 71549; Novagen, Madison, WI, USA), rabbit polyclonal anti‐nucleolin antibody (1 : 2000; ab22758, Abcam), or anti‐NS1 IgY, in PBS for 1 h at room temperature, washed with PBS twice, and incubated with secondary antibodies such as Alexa Fluor 405 goat anti‐rabbit IgG (1 : 2000; Invitrogen), Alexa Fluor 488 goat anti‐mouse IgG (1 : 2000; Invitrogen), Alexa Fluor 555 goat anti‐rabbit IgG (1 : 2000; Invitrogen), and FITC goat anti‐chicken/turkey IgG (1 : 100; Invitrogen), Alexa Fluor 568‐conjugated goat anti‐chicken IgG antibody (1 : 2000; Molecular Probes), in PBS for 1 h at room temperature, and then incubated for 5 min with 4′, 6‐diamidino‐2‐phenylindole (DAPI) (final concentration 1 μg·mL^−1^; Invitrogen). Cells were washed with PBS twice and water twice. After coverslips were dried, they were mounted on glass slides with Vectashield mounting reagent (Vector Corporation, Burlingame, CA, USA) and examined by an inverted fluorescence confocal microscope (FV1000; Olympus, Tokyo, Japan). Images were acquired by olympus fv10‐asw 1.3 viewer software and processed by Adobe Photoshop. Cells were fixed in 1.875% formalin and permeabilized in 0.5% NP‐40, or the cells were fixed in cooled methanol in different fixation procedures experiment. To locate the nuclei and mitochondria in cells, samples were simultaneously stained with DAPI and MitoTracker Orange CMTMRos (final concentration 150 nm; Molecular Probes), respectively.

### *In vitro* protein labeling with FlAsH‐EDT_2_

The cellular extracts of virus‐infected MDCK cells were prepared as described in previous section of immunoblotting. Before FlAsH‐EDT_2_ (Invitrogen) was added, the extracts were treated with or without β‐mercaptoethanol (final concentration 35.8 mm; Merck) and boiled for 5 min. To test FlAsH‐EDT_2_ binding ability on the native NS1‐tc proteins, the extract was mixed with FlAsH‐EDT_2_ without any treatment. The labeling condition was set at 70 °C for 10 min with FlAsH‐EDT_2_ (final concentration 2 μm) and then further incubated at room temperature for 1 h. Finally, the extract was analyzed by SDS/PAGE on a 12% polyacrylamide gel. The protein/FlAsH complexes were visualized on the gel with an appropriate filter for FlAsH fluorescence (excitation at 480 nm and emission at 535 nm) in Kodak image station 2000MM system. The same gel could be further analyzed for the total protein by Coomassie Blue staining or the specific NS1 with anti‐NS1 antiserum on the immunoblot.

### FlAsH‐EDT2 labeling of virus‐infected cells

Biarsenical labeling with FlAsH‐EDT2 in live cells was performed according to the manufacturer's instructions provided in the TC‐FlAsH™ and TC‐ReAsH™ II in‐cell tc‐tag detection kit (Invitrogen). Briefly, MDCK cells were grown on glass coverslips in 24‐well plates and infected with WT or mutant viruses at MOI of 1 at 37 °C for 8 h. At 7 hpi, cells were labeled with FlAsH‐EDT2 (final concentration 2 μm) in 250 μL of Opti‐MEM (Invitrogen) at 37 °C for 1 h. Supernatant was then discarded, and cells were washed once in BAL (2, 3‐dimercapto‐1‐propanol) wash buffer (final concentration 125 μm, supplied in the kit; Invitrogen) in Hank's balanced salt solution at 37 °C for 10 min. Subsequently, cells were fixed in 4% paraformaldehyde and processed for staining with anti‐NS1 antiserum as described previously. For live cells detection, the cells were grown on 35‐mm glass bottom culture dish (MatTek Corporation, Ashland, MA, USA) and labeled with MitoTracker Orange (Invitrogen) at 37 °C for 20 min. The cells were then infected at MOI of 1 and incubated with Hoechst 33342 (final concentration 2.5 μg·mL^−1^; Invitrogen) and FlAsH‐EDT2 at 37 °C for 1 h, then washed in BAL wash buffer as described above. Supernatant was discarded, and cells were incubated at 37 °C in a complete growth medium. Cells were then visualized under an inverted fluorescence confocal microscope (FV1000; Olympus) directly.

## Results

### NS1 protein localized at mitochondria in transfected cells

**Figure 1:**
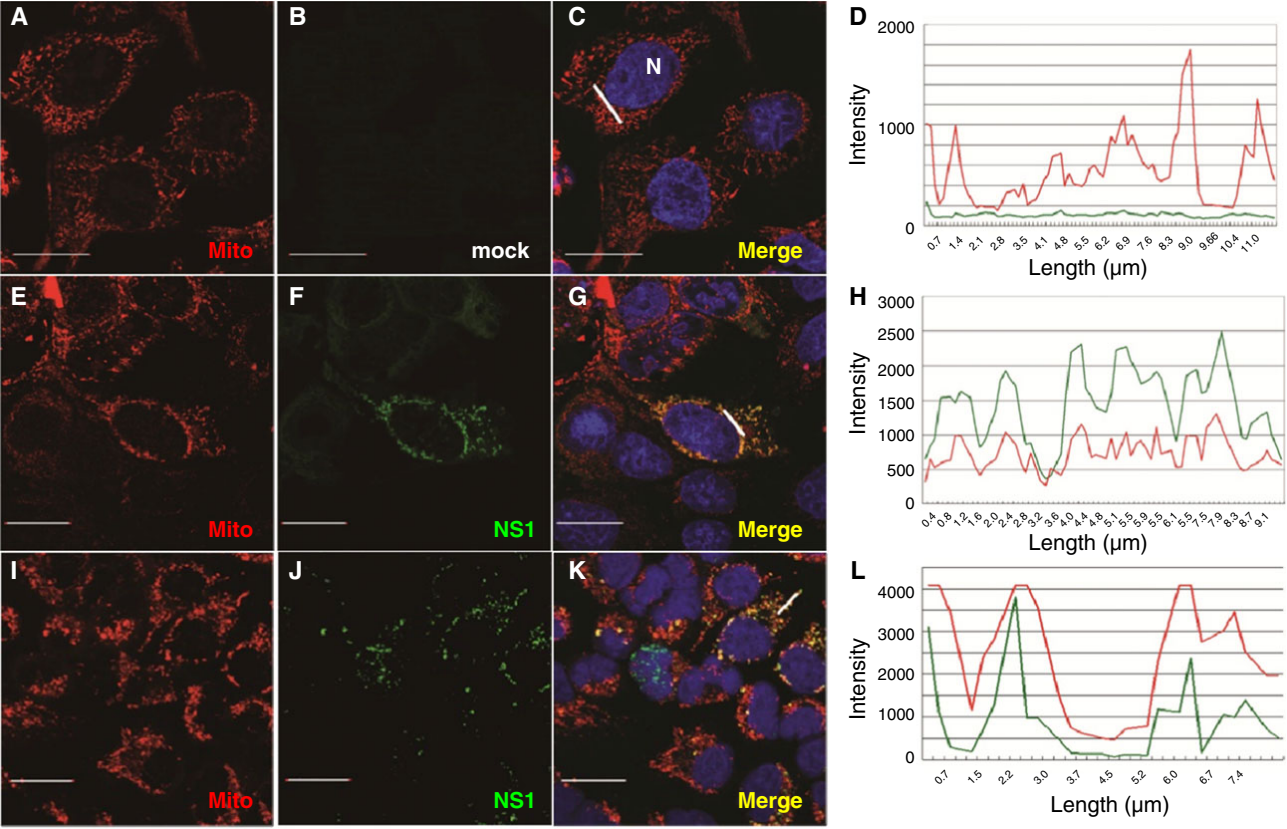
Mitochondrial localization of NS1 in transfected MDCK and A549 cells. MDCK (E–G) and A549 (I–K) cells were transfected with a plasmid encoding S‐tagged NS1 followed by MitoTracker Orange labeling and IFA using antibody against S‐tag. Mock‐transfected MDCK (A–D) serves as a negative control for NS1 expression. Nucleus was detected by DAPI staining. An overlay of the DAPI, MitoTracker, and NS1 signals is included (C, G, and K). An intensity plot over a cellular distance (white line indicated in panels C, G, K) of these merged images is shown (D, H, and L). In all panels, the scale bar represents a distance of 20 μm.

The subcellular localization of NS1 was initially examined in a transient expression system (Fig. 1). It was apparent that the signal for NS1 colocalized with the mitochondrial MitoTracker Orange in a subset of transfected MDCK (Fig. 1G); overall, mitochondria and nucleus localization can be found in 41% (40 of 98) and 34% (33 of 98) of transfected MDCK cells, respectively. However, 25% of transfected cells expressed NS1 in neither of these regions. Consistent results were observed in A549 cells (Fig. 1K). As a further indicator of colocalization, the signal intensities across a distance (represented by white lines in the merged images) from NS1 protein and MitoTracker Orange were plotted together. In contrast with the NS1‐mock‐transfected cells (Fig. 1D), the mirroring of peaks in intensity between the NS1 protein and MitoTracker Orange signal confirms the colocalization of both signals (Fig. 1H,L for MDCK and A549, respectively).

**Figure 2:**
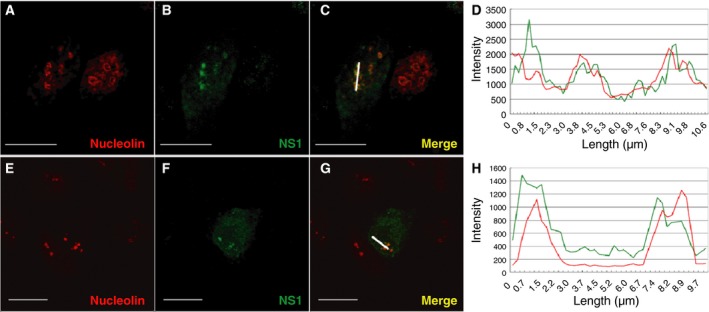
Nucleolar localization of NS1 in transfected MDCK and A549 cells. MDCK (A–D) and A549 (E–G) cells were transfected with a plasmid encoding S‐tagged NS1. Nucleolar localization of NS1 was detected by double staining with anti‐nucleolin and anti‐S‐tag antibodies. An overlay of the nucleolin and NS1 signals is included (C, G). An intensity plot over a cellular distance (white line) of these two merged images is analyzed and shown (D, H). In all panels, the scale bar represents a distance of 20 μm.

In addition to mitochondria targeting, nucleolar localization pattern was also noted in a subset of transfected cells (Fig. 2) that was further confirmed by IFA with an anti‐nucleolin antibody. It was apparent that the signal for NS1 colocalized with the nucleolar signal of MDCK cells (Fig. 2C,D). To determine whether this distribution of NS1 was not only specific to the MDCK cell line, the S‐NS1A plasmid was transfected into A549 cells, followed by IFA (Fig. 2G,H).

### Infectious recombinant influenza A virus with a tc‐tag in NS1 linker region or effector domain

To confirm the NS1 mitochondria localization and to avoid the possible artifact resulted from procedure(s) of IFA, we visualized the dynamic localization of NS1 during virus infection in live cells. Taking advantage of fluorescent labeling of tc‐tagged proteins, we generated recombinant IAV PR8 strain expressing NS1 with insertion of tc‐tag. As NS2/NEP mRNA is derived from NS1 mRNA by splicing, part of the coding region in NS1 mRNA is the intron of the NS2/NEP mRNA. Therefore, the sequence encoding short peptide tc‐tag (CCPGCC) introduced into NS1 at the intron region of NS2/NEP mRNA will not alter the structure and functions of NS2/NEP. Previous report showed that the linker region between RNA‐binding domain and effector domain, and the loop region in the effector domain of NS1 are the two most suitable sites for inserting a tc‐tag according to the reported X‐ray structure of NS1 13, 39. Hence, the tc‐tag sequence (as indicated in bold and underline) was inserted in the corresponding region (Table 1), and the resultant mutant viruses were designated as PR8/NS1‐tc‐linker and PR8/NS1‐tc‐ED, respectively.

**Table 1:** Sequences and location of tc‐tag insertion on NS1 gene

Virus	NS1 protein sequence	Comment
PR8 wt	E_75_ALKMTMASV_84_	tc‐tag, shown in bold and underline, in NS1 protein linker region at residue 79
NS1‐tc‐linker	EALKM**CCPGCC**TMASV	
PR8 wt	Q_121_AIMDK_126_	tc‐tag in NS1 protein effector domain at residue 123
NS1‐tc‐ED	QAI**CCPGCC**MDK	

**Figure 3:**
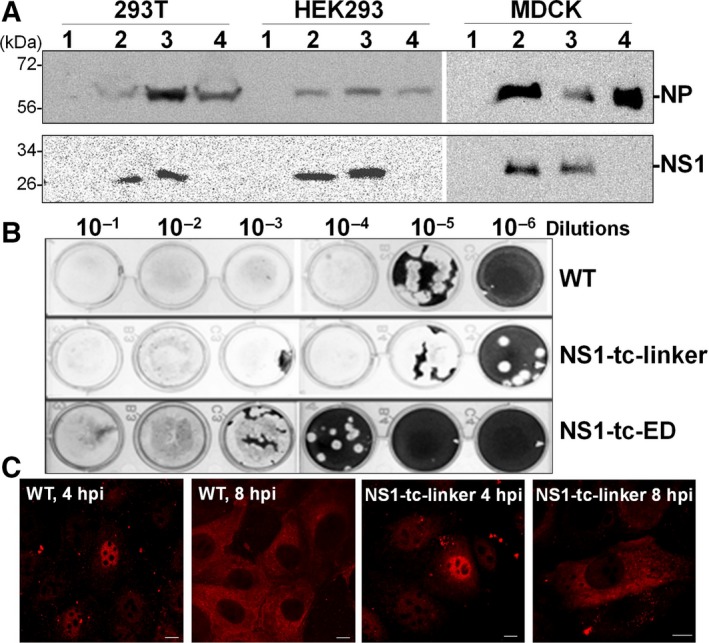
Generation of IAV mutants expressing tc‐tagged NS1 protein. (A) Recombinant influenza viruses were generated by reverse genetics and the expression of viral NP and NS1 proteins from 293T, HEK 293, and MDCK cells that were mock‐infected (lane 1) or infected with PR8 (lane 2), NS1‐tc‐linker virus (lane 3), or NS1‐tc‐ED virus (lane 4) were detected by immunoblotting. (B) Plaque assay was used to measure the infectivity of these influenza viruses including WT (strain of PR8) and two NS1‐mutant viruses (NS1‐tc‐linker virus and NS1‐tc‐ED virus). (C) The localization of NS1 at 4 and 8 hpi of WT or NS1‐tc‐linker virus in MDCK cells. In all panels, the scale bar represents a distance of 10 μm.

As shown in Fig. 3A, infection of PR8 (WT), PR8/NS1‐tc‐linker, and PR8/NS1‐tc‐ED accumulated similar levels of NP proteins in various cell lines like MDCK, 293T, and HEK293, but only PR8 and PR8/NS1‐tc‐linker viruses show detectable levels of NS1 expression. In addition, as with WT (PR8), both the PR8/NS1‐tc‐linker and PR8/NS1‐tc‐ED viruses would form distinct plaques, although the yield of PR8/NS1‐tc‐ED virus progeny is lower than that of the other two viruses (Fig. 3B). These results suggest that both mutants can generate viable recombinant viruses, but the insertion of tc‐tag in the effector domain may have adverse effects on the NS1 expression. Next, we further examined whether the presence of tc‐tag affects cellular distribution of NS1 by IFA in comparison with that of WT. Results show that the NS1 derived from NS1‐tc‐linker virus shares similar localization patterns to those of WT‐infected cells. NS1 proteins predominantly localize in the nucleus at 4 hpi and in the cytoplasm and nucleus at 8 (Fig. 3C).

### NS1 derived from PR8/NS1‐tc‐linker virus successfully labeled with FlAsH

**Figure 4:**
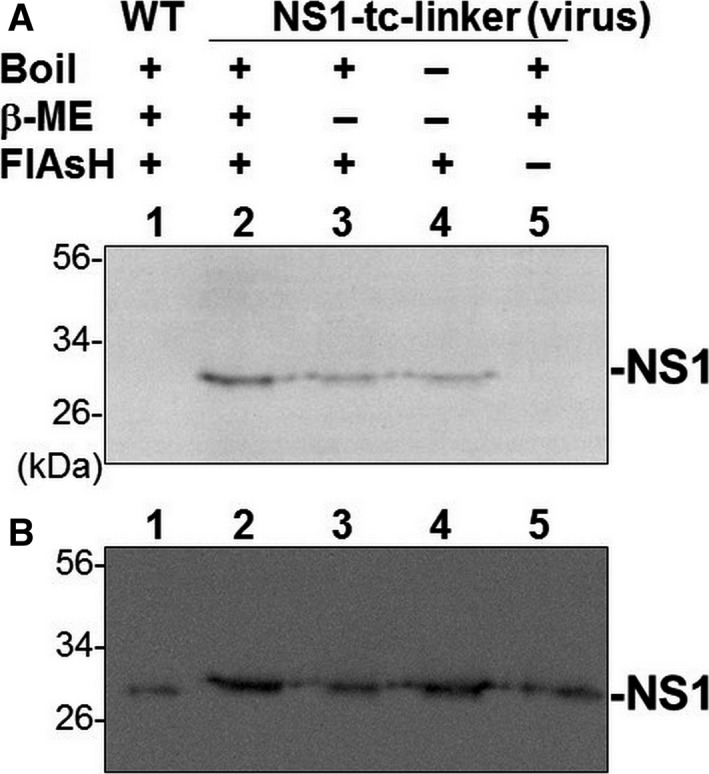
*In vitro* labeling of NS1‐tc‐linker proteins with FlAsH. (A) After infection of WT or NS1‐tc‐linker virus (lanes 1–4) for 18 h, MDCK cells were lysed and cell lysates were incubated with FlAsH. The treatment of each sample was shown above each lane. The FlAsH signal was visualized on the gel with appropriate filters for FlAsH fluorescence (excitation at 480 nm and emission at 535 nm) using Kodak image station 2000MM system. (B) NS1 expression was detected by immunoblot analysis.

To test whether the NS1 produced from NS1‐tc‐linker virus could bind FlAsH, we performed an *in vitro* labeling experiment. As the accumulation of NS1 of PR8/NS1‐tc‐ED virus is under detection level (Fig. 3A), we performed all follow‐up experiments using only the PR8/NS1‐tc‐linker virus. The extracts derived from PR8/NS1‐tc‐linker‐infected cells were incubated with FlAsH and analyzed on a SDS/PAGE. We could directly visualize the FlAsH fluorescence emitted from the NS1‐tc‐linker–FlAsH complexes on the gel after excitation at 480 nm (Fig. 4A). The results also indicate that the denatured form of the NS1‐tc‐linker protein can interact with FlAsH more efficiently in the presence of β‐mercaptoethanol than those without β‐mercaptoethanol treatment and in its native form (Fig. 4A). Immunoblot analysis with anti‐NS1 antiserum confirms that NS1 proteins were indeed produced from WT and PR8/NS1‐tc‐linker virus and all were of expected size (Fig. 4B). These results indicate that NS1‐tc‐linker proteins can specifically bind FlAsH *in vitro* and labeling efficiency can be enhanced in the presence of a reducing agent β‐mercaptoethanol. These results also suggest that NS1‐tc‐linker can be labeled with FlAsH in a live cell either during the translation process or in a reduced condition inside certain organelles.

### NS1‐tc‐linker protein localized at mitochondria during early infection

**Figure 5:**
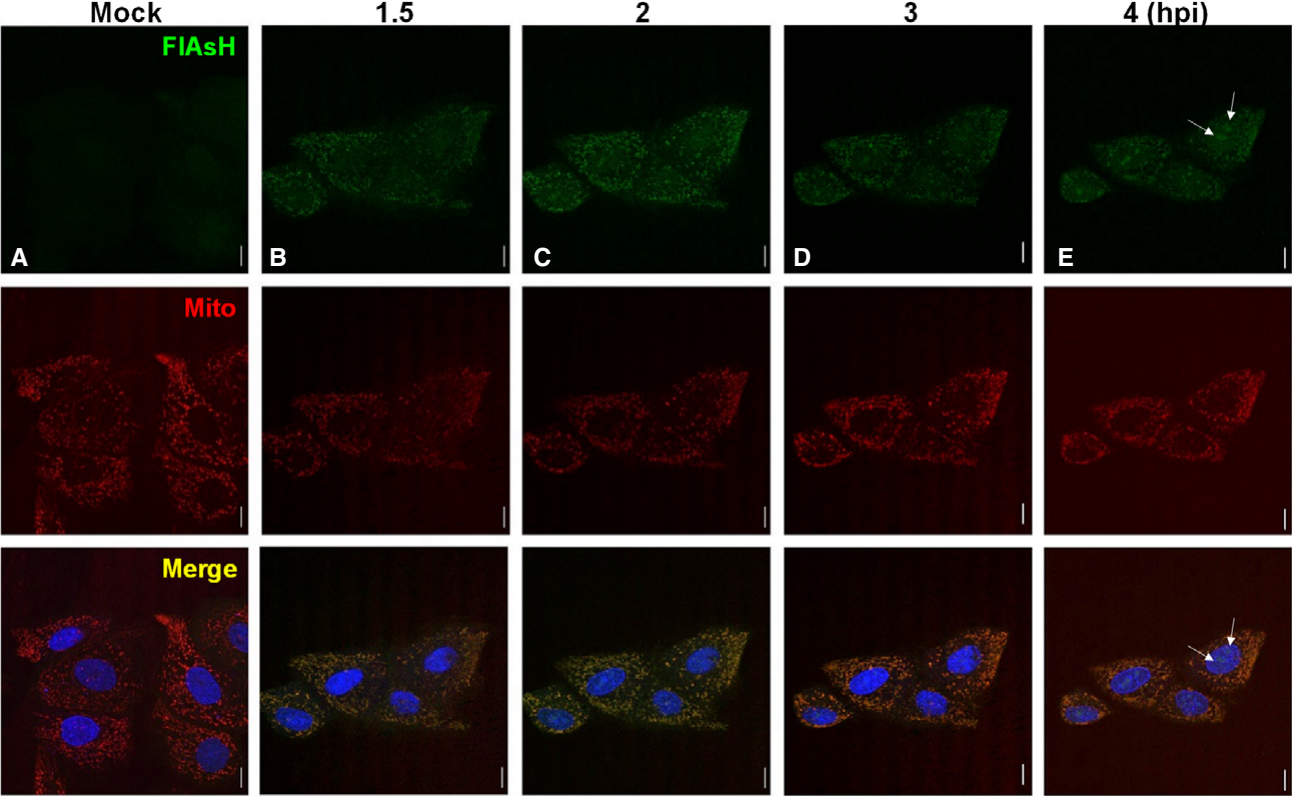
The localization of NS1‐tc‐linker protein was detected in live cells at 1.5 hpi after FlAsH labeling. MDCK cells were infected with NS1‐tc‐linker viruses for 1 h, then incubated with 1 μm FlAsH at 37 °C for 20 min, and washed by BAL buffer for 10 min. The nucleus was labeled with Hoechst, and the mitochondria were labeled with Mito Tracker Orange. The signals were visualized from the same cells at 1.5, 2, 3, and 4 hpi (panel B–E). In all panels, the scale bar represents a distance of 10 μm, and panel (A) indicates image of mock‐infected cells. Merge indicates superimposition of images of FlAsH and Mito.

To inspect the localization of the NS1‐tc‐linker protein during virus infection in live cells, MDCK cells were infected with PR8/NS1‐tc‐linker and simultaneously incubated with FlAsH for 1 h. After removing free FlAsH from the medium, we observed the FlAsH signal in a subcellular organelle that colocalized with the staining of mitochondrial Mito Tracker Orange at the earliest time point of 1.5 hpi (Fig. 5, panel B). We also traced the signal from the same cells for every hour until 4 h after labeling. This study finds that the FlAsH signal presence in mitochondria lasts up to 4 hpi (Fig. 5, panel B–E). The most intensive FlAsH signal in mitochondria can be observed at 2 hpi (Fig. 5, panel C) and starts fading afterward. Besides, we also observe the granular pattern formed in the nucleus at 3–4 hpi (Fig. 5D,E). Noticeably, NS1‐tc‐linker signal redistributed to nucleus and accumulated at distinct foci (Fig 5, panel E, indicated with arrows). These results suggest that NS1‐tc‐linker produced at the first hour after infection (FlAsH labeled) was targeted to mitochondria. The accumulation of signals reaches a maximum at about 2 hpi and then fades (possibly due to degradation through time) or translocates from the mitochondria into subnuclear domains.

**Figure 6:**
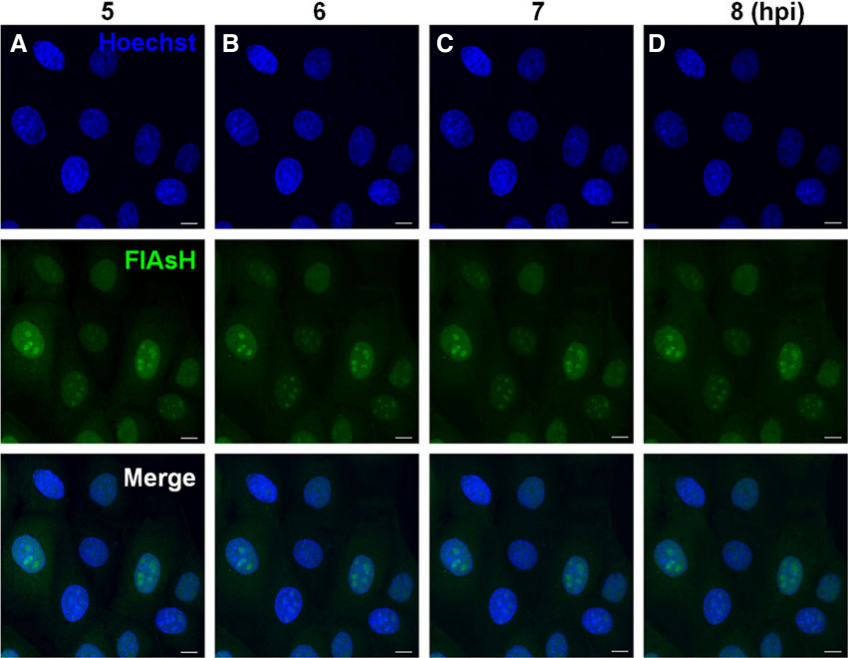
The localization of NS1‐tc‐linker protein was detected in live cells at later time point of infection after FlAsH labeling. MDCK cells were infected with NS1‐tc‐linker viruses for 3 h, then incubated with 1 μm FlAsH at 37 °C for 1 h, and washed by BAL buffer for 10 min. The nucleus was labeled with Hoechst, and the mitochondria were labeled with Mito Tracker Orange. The signals were visualized in the cells infected with NS1‐tc‐linker virus at 5–8 hpi with every 1‐h intervals. In all panels, the scale bar represents a distance of 10 μm.

The possible relocalization of NS1 from mitochondria to nucleus at an early time of infection (Fig. 5, panels B–E) led us to examine whether newly synthesized NS1‐tc‐linker protein at 3–4 hpi would localize in the mitochondria or was already in the nucleus. Three hours after PR8/NS1‐tc‐linker virus infection, MDCK cells were incubated with FlAsH for 1 h. We detected that the FlAsH signal was stronger in the nucleus than in the cytoplasm; nevertheless, the granular pattern forms clearly in the nucleus at 5 hpi (Fig. 6, panel A). The granular structure patterns in the nucleus are maintained persistently till 8 hpi (Fig. 6, panel D). These results indicate that NS1‐tc‐linker proteins synthesized at 3–4 hpi are transported into the nucleus and remain in the nucleus until at least 8 hpi, whereas some of the NS1 synthesized at this time point is located in the cytoplasm but does not transport into the mitochondria.

## Discussion

In this study, cellular trafficking of NS1 was monitored by means of a recombinant influenza viruses expressing tc‐tag‐fused NS1 protein after infection in live cells (Fig. 3A). Upon FlAsH labeling, the newly synthesized NS1 localized in mitochondria at very early time of infection (1.5 hpi; Fig. 5B). Shortly thereafter, NS1 proteins are mostly localized in the nucleus and form granular patterns at 3–4 hpi (Fig. 5). At later infection time (7–8 hpi), NS1 proteins are predominantly localized in the nucleus, but some can be detected in the cytoplasm (Fig. 6).

Previously, Li *et al*. 27 monitored NS1 subcellular localization using the same strategy, of which TC‐tag was inserted at the helix region or loop/linker regions of PR8 NS1. It was shown that loop/linker, rather than helix region, is suitable site for the insertion of a short tag. Consistently, the cytoplasm–nucleus shuttling was observed during 5–6 hpi 27. However, mitochondria localization of NS1 with TC inserted at the same position as PR8/NS1‐tc‐linker virus was not noticed in their study. This is possibly due to the experimental design and the timing of images acquired. FlAsH labeling condition was optimized that allows us to detect expression of NS1 as early as 1.5 hpi. In their study, FlAsH was added at 4 hpi and images were then obtained at 3‐min intervals, up to 171 min (~ 7 hpi). Hence, most likely, timing of mitochondria targeting (1.5–4 hpi) has been missed.

**Figure 7:**
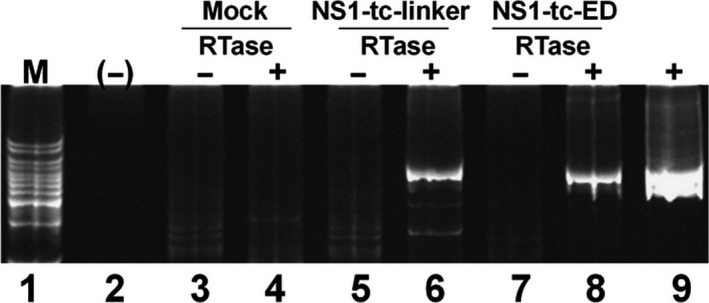
The measurement of NS1 mRNA expression by RT‐PCR. The total RNAs of virus‐infected MDCK cells was reverse‐transcribed by SuperScript™ III with reverse transcription (RT) primer. The cDNA fragments were then amplified by PCR with NS1‐specific forward primer and reverse primer. The PCR products were cloned into pGEM‐T Easy vector and sequenced. The negative control of RT (without adding RTase) is indicated in lanes 3, 5, and 7. The negative and positive controls of PCR are indicated in lane 2 and lane 9, respectively.

To generate a NS1‐traceable virus, the function of NS1 protein has to remain unaffected. Two sites were considered for tc‐tag insertion, in the linker region (at residue 79) and in the effector domain (at residue 123). However, only the PR8/NS1‐tc‐linker virus efficiently expresses mutant NS1 in infected MDCK cells or transfected 293 cells (Fig. 3). This is consistent with the Li *et al*.'s 27 study, which proposed that the linker region (residues 74–79) appears to be an ideal position to accommodate the insertion of a short tag without altering NS1 function. The defect in the accumulation of NS1 protein derived from PR8/NS1‐tc‐ED is possibly due to the instability of the NS1 protein derived from PR8/NS1‐tc‐ED, as the expression level of NS1‐tc‐ED mRNAs is similar to that of WT (Fig. 7). Hence, we assume the failure of rescuing NS1‐tc‐ED virus is due to the misfolding of NS1‐tc‐ED protein that possibly results in rapid degradation.

Supported by a previous study 40, an *in vitro* FlAsH labeling experiment shows that NS1‐tc‐linker requires a reduced condition for efficient labeling (Fig. 4A), indicating that labeling of NS1‐tc‐linker with FlAsH most likely occurs during the translation process. Therefore, the signal we have detected is the newly synthesized NS1‐tc‐linker proteins pulse‐labeled at the time of FlAsH treatment. However, after labeling, the signals might reside persistently at the location after the first visualization. The signal localized in the mitochondria remains for at least 4 h once synthesized right after infection. Similarly, the FlAsH signal formed the granular pattern in nucleus observed at 5 and 8 hpi (Fig. 6).

Localization of NS1 in the mitochondria has not yet been reported in influenza viruses. The only influenza viral proteins reported to localize in mitochondria are PB2 and PB1‐F2 4, 41. Influenza virus infection leads to cell apoptosis that may be due to the localization of PB1‐F2 in mitochondria 42, 43 and the active form of Bax, a pro‐apoptotic protein of the Bcl‐2 family, translocated from cytoplasm to mitochondria 44. Efficient induction of apoptosis and virus replication requires Bax activation. However, we observed NS1 localized in mitochondria at the very early time point of infection (at 1.5 hpi), but not at other later time points (from 4 to 8 hpi). The possible role of NS1 located in mitochondria as early as 2 hpi is to protect infected cells from early apoptotic cell death, which allows for longer virus progeny production. Influenza virus infection triggers apoptosis by intrinsic and extrinsic mechanisms. NS1 is one of the best‐studied influenza virus inducers 45. Several lines of evidence have indicated that NS1 inhibits apoptosis via interaction of the regulatory subunit p85 of phosphatidylinositol 3‐kinases (PI3Ks) and activates the PI3K/Akt pathway 32, 33. However, whether the localization of NS1 to mitochondria coincides with its function on regulation of apoptosis or other cellular processes requires further consideration.

Interestingly, since the identification of mitochondrial antiviral‐signaling protein (MAVS), mitochondria gained increasing attention for its role on innate immunity modulation. MAVS, serving as a critical adaptor, mediates signaling pathway for the production of type I IFNs (IFN‐I) 46. Upon virus infection, viral RNA binds retinoic acid‐inducible gene I (RIG‐I)‐like receptors, such as RIG‐I and melanoma differentiation‐associated protein 5, which in turns activates its sequel signaling, including translocation of RIG‐I to mitochondria where RIG‐I binds MAVS and ultimately triggers MAVS‐mediated IFN expression signaling pathway 47. It has been shown that IAV NS1 suppresses RIG‐I/MAVS signaling axis by interacting with RIG‐I 34 and the tripartite motif‐containing protein 25, an E3 ubiquitin ligase that catalyzes RIG‐I ubiquitination crucial for its downstream signaling 36. NS1 does not harbor mitochondria targeting sequences, and therefore, distribution of NS1 to mitochondria relies on MAVS and whether such a spatial coincidence enhances the inhibitory effect of NS1 on IFN production is worthy of investigation.

In summary, as indicated in other research studies, a mutant virus with tc‐tagged NS1 exerts an indistinguishable phenotype as WT influenza virus 27. Here, we further optimized this biarsenical labeling system for real‐time monitoring the intracellular trafficking of NS1 in live cells at early time points of influenza virus infection. Most interestingly, we detect NS1 and its novel mitochondria localization as early as 1.5 hpi. In addition, the dynamic distribution, that is, translocation from mitochondria to nucleus and later relocation to cytoplasm, is demonstrated clearly in this system. The novel observation on NS1 mitochondria targeting sheds some light on how the NS1 protein exerts the dynamic multitask on apoptosis and counteracting innate antiviral response, and also a new strategy that IAV employed for efficient infection.

## Author contributions

W‐LH and C‐HT designed the experiment, interpreted results, and finalized the manuscript. C‐FT conducted the majority of experiments and also wrote the draft of the manuscript. H‐YL performed the experiments for Figs 1 and 2.
